# Intrinsic pathway activation in patients with antiphospholipid syndrome and healthy controls

**DOI:** 10.1016/j.rpth.2025.102694

**Published:** 2025-01-31

**Authors:** Dagmar J.M. van Mourik, Valérie L.B.I. Jansen, Michiel Coppens, Saskia Middeldorp, Hugo ten Cate, Harry R. Büller, Henri M.H. Spronk, Magdolna Nagy, Thijs E. van Mens

**Affiliations:** 1 Department of Vascular Medicine, Amsterdam UMC, University of Amsterdam, Amsterdam, the Netherlands; 2 Amsterdam Cardiovascular Sciences, Pulmonary Hypertension and Thrombosis, Amsterdam, the Netherlands; 3 Division of Thrombosis and Hemostasis, Department of Internal Medicine, Leiden University Medical Center, Leiden, the Netherlands; 4 Amsterdam Reproduction and Development, Amsterdam, the Netherlands; 5 Department of Internal Medicine, Radboud University Medical Center, Nijmegen, the Netherlands; 6 Department of Internal Medicine, Maastricht University Medical Center, Maastricht, the Netherlands; 7 Department of Biochemistry, Maastricht University Medical Center, Maastricht, the Netherlands

**Keywords:** antiphospholipid syndrome, coagulation cascade, factor XII, intrinsic pathway, thrombotic autoimmune disease

## Abstract

**Background:**

Antiphospholipid syndrome (APS) is a thrombotic autoimmune disease. Activation of the intrinsic coagulation pathway contributes to inflammatory and cardiovascular diseases, but its role in APS is unknown. Increased release of neutrophil extracellular traps and reduced effectiveness of direct oral anticoagulants support the hypothesis of increased intrinsic pathway activation in patients with APS, which is relevant considering the ongoing development and clinical testing of intrinsic pathway inhibitors.

**Objectives:**

To compare *in vivo* intrinsic pathway activation of patients with APS and healthy controls.

**Methods:**

Patients with APS without recent thrombotic or obstetric events and healthy controls were investigated. ELISAs were used to measure activated coagulation factors in complex with the natural inhibitors antithrombin or C1-esterase inhibitor in plasma. The primary outcome of this study was factor (F)XII activation, which initiates the intrinsic pathway. Secondary outcomes included activation of downstream intrinsic coagulation FXI and FIX.

**Results:**

Plasma of 73 patients with APS and 19 healthy controls showed no significant difference in activated FXII-inhibitor complexes. The concentrations of activated FXI and FIX and inhibitor complexes likewise did not differ between the groups. A subanalysis of patients with APS by anticoagulant use showed no difference for FXII and FXI activation.

**Conclusion:**

Intrinsic pathway activation in patients with APS without recent thrombotic or obstetric events did not differ significantly compared with healthy controls.

## Essentials

• Patients with thrombotic antiphospholipid syndrome (APS) respond poorly to direct oral anticoagulants.
• Inhibitors against intrinsic pathway factors XI and XII are in development.
• A cohort study assessed intrinsic pathway activation in patients with APS and healthy controls.
• Outside of thrombotic episodes, patients with APS show no increased intrinsic pathway activation.

## 1 Introduction

Antiphospholipid syndrome (APS) is an autoimmune disease defined by the occurrence of obstetric morbidity and/or thromboembolic events in the persistent presence of antiphospholipid antibodies [1]. The thrombotic events can occur throughout the vasculature, in veins, in arteries, or in the microcirculation. Antiphospholipid antibodies target phospholipids and phospholipid-binding proteins, such as cardiolipin and β2 glycoprotein (GP)1 [2]. Although antiphospholipid antibodies have a wide range of effects on the hemostatic and fibrinolytic system and affect various cells including endothelial cells, neutrophils, and platelets, which of these mechanisms is predominant in causing the prothrombotic state in patients with APS is yet to be established [[3], [4], [5]]. Accumulating evidence indicates that activation of the intrinsic pathway contributes to inflammatory and cardiovascular diseases, such as severe COVID-19 infection, sepsis, coronary heart disease, and stroke [[6], [7], [8], [9]]. The intrinsic pathway is initiated upon factor (F)XII activation by prekallikrein, high-molecular-weight kininogen or through contacting negatively charged surfaces [10]. Whether the intrinsic pathway also contributes to the prothrombotic state in APS has not been studied.

Two observations could accord with increased intrinsic pathway activation in APS. One is the role of neutrophil extracellular traps (NETs) in APS pathophysiology [11]. Anti-β2GP1 antibodies as well as plasma from patients with APS have been shown to induce NETosis, that is, the release of NETs, in neutrophils of healthy controls [12,13]. NETs produce negatively charged surfaces, which can trigger activation of the intrinsic pathway in mice *in vivo* [14]. Factor XII is indeed able to bind to the negatively charged surfaces of NETs, which serves as a scaffold for FXII activation and, thereby, may initiate the intrinsic pathway [14]. Second, several randomized controlled trials have shown that direct oral anticoagulants (DOACs) are less effective in preventing recurrent thrombotic events, especially stroke, in patients with APS compared with vitamin K antagonists (VKAs) [[15], [16], [17]]. DOACs specifically target the common pathway by inhibition of activated FXa or by thrombin. In contrast, VKAs deplete not only the FII, FVII, and FX but also FIX, thereby inhibiting the intrinsic pathway. A more prominent contribution of the intrinsic pathway to thrombosis in patients with APS could be an explanation for the lower effectiveness of DOACs than that of VKAs in this population. This is in line with observed lower effectiveness of DOACs in patients with mechanical heart valves [18,19]. Clinical trials with dabigatran and apixaban were terminated early due to excess thrombotic and bleeding events in these patients compared with that in patients treated with VKA. These disappointing results have been attributed to the negatively charged surface on the mechanical heart valve that constantly activates FXII, which initiates the intrinsic pathway.

Evidence of increased intrinsic pathway activation is of importance since the emerging FXI inhibitory agents may provide an alternative treatment for patients with APS. Three types of FXI inhibitors are currently studied in phase II and III trials: antisense oligonucleotides, monoclonal antibodies, and small molecules [20,21]. Although safety and efficacy data from phase III trials will have to be awaited, the pathophysiologic role of NETosis in APS and its potential for activating FXII along with the disappointing efficacy of DOACs support the exploration of the role of the intrinsic pathway in APS.

Recent advances in measurement techniques allow for quantification of *in vivo* intrinsic pathway coagulation factor activity [22,23]. We aimed to evaluate intrinsic pathway activation in APS. *In vivo*–activated coagulation factors can be assessed by measuring them in complex with their natural inhibitors. We aimed to quantify these enzyme-inhibitor complexes in patients with APS and healthy controls [7,22,23].

## 2 Methods

### 2.1 Subjects and study design

We included patients with APS diagnosed according to the 2006 Sydney classification criteria without thrombotic or obstetric events in the last year [1], from the Amsterdam APS Biobank. Patients with APS were eligible for inclusion irrespective of their APS type, including primary and secondary APS, as well as, thrombotic and/or obstetric APS. Antibody profiles were not determined at inclusion; instead, historical profiles were used; historical antibody titers were measured with EliA kits from Thermo Fisher with the 99th percentile as cutoff for positivity. Patients were recruited through the outpatient clinic of our tertiary care vascular medicine department and were included regardless of use of anticoagulant therapy. We included patients and healthy controls at a single time point between November 2016 and December 2021. Healthy controls were recruited through APS participants, from the social environment of the included patients, excluding family or household members. General health data and APS relevant comorbidities were recorded for patients and healthy controls alike. Inclusion was not formally consecutive as it depended on the treating physician, but an effort was made to include all eligible subjects. All participants provided their written informed consent. The study was approved by the Amsterdam UMC Biobank Committee and conducted in agreement with the tenets of the Declaration of Helsinki. Blood was drawn via venipuncture using a tourniquet, and general measures were taken to prevent long stasis time. The blood was collected in plastic citrate and EDTA tubes.

The primary outcome of this study was FXII activation, since NETosis is hypothesized to induce this activation and because FXII activation is not affected—as opposed to downstream coagulation factors—by thrombin generation, which in turn is also influenced by treatment [14]. Activated FXII was determined in complex with its natural inhibitors (antithrombin [AT] and C1-esterase inhibitor [C1Inh]), with 2 complementary assays. For a comprehensive overview of the role of intrinsic pathway activation in APS pathogenesis, activated FXI and FIX complexes were determined as secondary outcomes.

### 2.2 Assays

Activated FXIIa-AT and FXIIa-C1Inh complexes were measured in EDTA plasma as previously described with minor modifications [22,23]. We used F3 antibody directed against FXII (2 μg/mL) [22] or KOK12 antibody directed against C1Inh (1.5 μg/mL) [22] as capture antibodies. We applied biotinylated sheep antihuman AT III antibody (1-1.5 μg/mL; Affinity Biologicals) or biotinylated F3 antibody directed against FXII (1 μg/mL) [22] as detection antibodies.

Further, coagulation activity was measured by detection of activated coagulation factors, FXIa and FIXa, in complex with their natural inhibitors (AT and/or C1Inh) using 3.2% sodium citrate anticoagulant plasma as previously described with minor modifications [22,23]. In brief, ELISA plates were coated with sheep antihuman FXI antibody (XI-5, 2.5 μg/mL) [22], and antihuman FIX antibody (2 μg/mL; Affinity Biologicals). Detection of the inhibitor complexes was done through biotinylated sheep antihuman AT III antibody (1-2.5 μg/mL; Affinity Biologicals) or biotinylated monoclonal antibody R11 directed against C1Inh (1.5 μg/mL) [22]. Enzyme-inhibitor complex levels in the plasma samples were calculated using reference curves consisting of known amounts of respective enzyme-inhibitor complexes.

### 2.3 Analysis

Baseline characteristics were summarized with descriptive statistics. For all coagulation factors we compared enzyme-inhibitor complex levels between patients with APS and healthy controls. We performed subanalyses in the APS cohort for anticoagulant medication use, to observe the effect of anticoagulants on the activity of intrinsic coagulation factors, and phenotype of APS (thrombotic or obstetric). In a post hoc analysis, we assessed FXII complexes in the subgroup of patients with APS and systemic lupus erythematosus (SLE). Assay measurements below the lower limit of detection were imputed with half the value of the lower limit of detection. Measurements above the higher limit of detection were imputed with the value of the higher limit of detection. Values were log-transformed to achieve normal distribution and analyzed with an independent *t*-test. *P* values of <.05 were considered statistically significant. All data were analyzed in SPSS statistics, version 29, and figures were created in GraphPad Prism, version 9.3.1.

## 3 Results

In total, 73 patients with APS and 19 healthy controls were included (Table). The mean age was similar in both groups. There was an overrepresentation of women in both patients with APS and healthy controls; 41 patients with APS used anticoagulants (23 VKA, 17 DOAC, and 1 low molecular weight heparin), compared with none of the healthy controls.

**Table:** Baseline characteristics.

Characteristic	Patients with APS (*n* = 73)	Healthy controls (*n* = 19)
Age (y), mean ± SD	40.1 ± 10.0	40.4 ± 12.4
Female sex, *n* (%)	66 (90.4)	19 (100.0)
BMI, median (IQR)	25.7 (22.7-28.7)	23.4 (21.8-27.8)
Ethnicity, *n* (%)		
Caucasian	62 (84.9)	19 (100.0)
Creole	2 (2.7)	—
Mediterranean	1 (1.4)	—
Mixed	2 (2.7)	—
Other	6 (8.2)	—
Current smoking, *n* (%)	13 (17.8)	4 (21.1)
SLE, *n* (%)	11/70 (15.7)	0 (0.0)
History of thrombosis, *n* (%)		
Venous thromboembolism	31 (42.5)	1 (5.3)
Arterial thromboembolism	28 (38.4)	0 (0.0)
History of pregnancy, *n* (%)	49/66 (74.2)	16 (84.2)
History of obstetric events, *n* (%)		
Late fetal death	32/49 (65.3)	2/16 (12.5)
≥3 early pregnancy losses	8/49 (16.3)	0/16 (0.0)
Preterm birth	2/49 (4.1)	0/16 (0.0)
Obstetric APS, *n* (%)	20 (27.4)	NA
Thrombotic APS, *n* (%)	37 (50.7)	NA
Obstetric and thrombotic APS, *n* (%)	16 (21.9)	NA
Antiphospholipid antibodies, *n* (%)		
Anti-β2GP1 IgG	36 (49.3)	NA
Anti-β2GP1 IgM	13 (17.8)	NA
Anticardiolipin IgG	45 (61.6)	NA
Anticardiolipin IgM	31 (42.5)	NA
Lupus anticoagulant	46 (63.0)	NA
Triple positive	8 (11.0)	NA
Anticoagulant use, *n* (%)		
DOAC	17 (23.3)	0 (0.0)
VKA	23 (31.5)	0 (0.0)
LMWH	1 (1.4)	0 (0.0)
None	32 (43.8)	19 (100.0)
Antiplatelet therapy, *n* (%)	16 (21.9)	0 (0.0)

APS, antiphospholipid syndrome; BMI, body mass index; DOAC, direct oral anticoagulant; GP, glycoprotein; LMWH, low molecular weight heparin; NA, not applicable; SLE, systemic lupus erythematosus; VKA, vitamin K antagonist.

### 3.1 FXII activation

**Figure 1:**
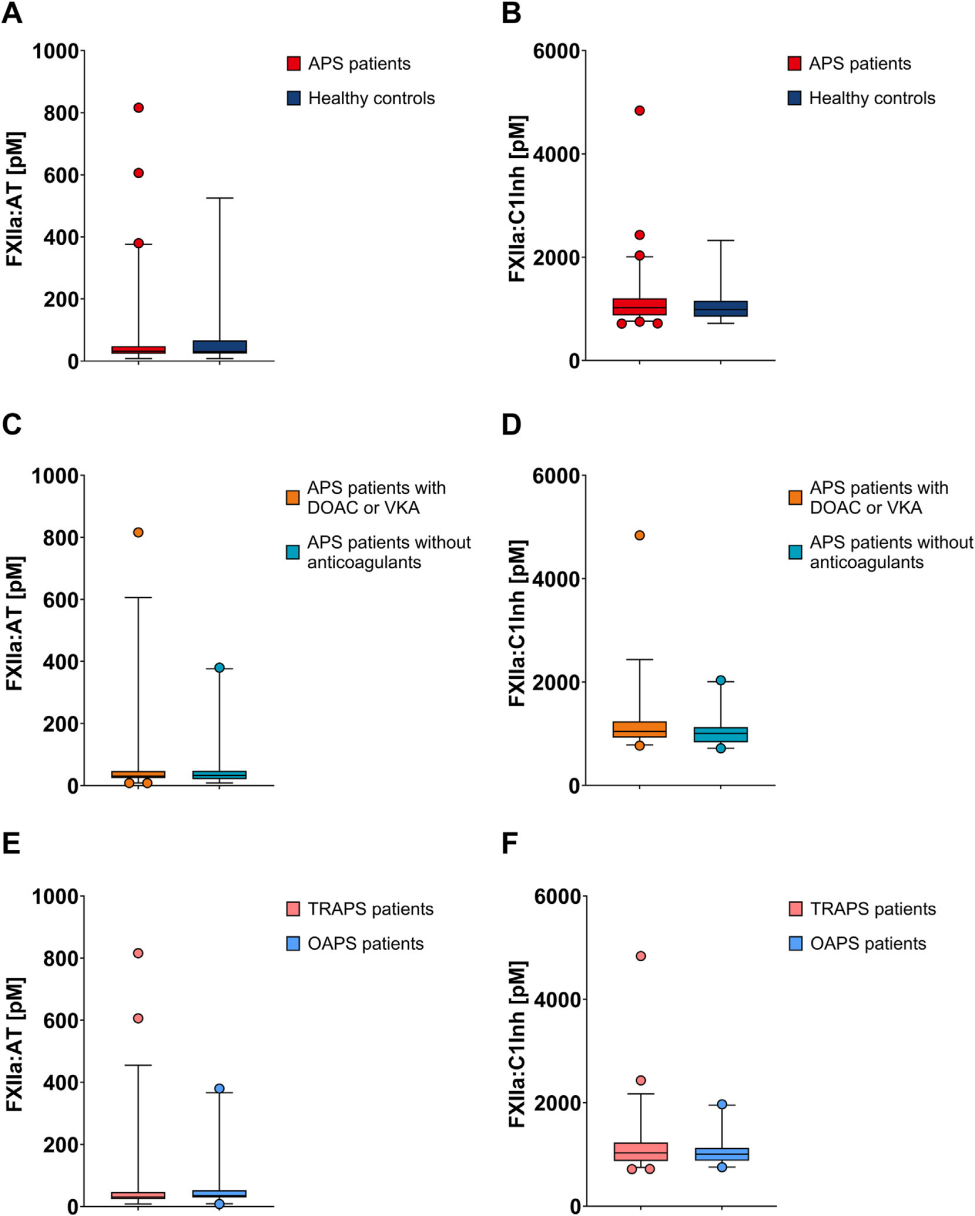
Factor (F)XII activation. Activated FXII was determined in complex with antithrombin (FXIIa-AT) (A) and C1-esterase inhibitor (FXIIa-C1Inh) (B) in patients with antiphospholipid syndrome (APS; *n* = 72) and healthy controls (*n* = 19). A subanalysis measured FXIIa-AT (C) and FXIIa-C1Inh (D) in patients with APS using direct oral anticoagulants (DOACs) or vitamin K antagonists (VKAs; *n* = 39) and in patients with APS without anticoagulants (*n* = 32). A second subanalysis determined FXIIa-AT (E) and FXIIa-C1Inh (F) in thrombotic patients with APS (TRAPS; *n* = 52) and in obstetric patients with APS (OAPS; *n* = 20). Data are expressed as median with interquartile range. For statistical analysis, the data were log-transformed and analyzed using an independent *t*-test.

Factor XII activation did not differ between patients with APS and healthy controls. Median FXII activation as measured by FXIIa-AT was 32.0 pM (IQR: 23.8-48.1 pM) in patients with APS and 30.5 pM (IQR: 24.7-66.8 pM) in healthy controls (*P* = .782) (Figure 1A). Median FXII activation as measured by FXIIa-C1Inh was 1021.6 pM (IQR: 874.7-1203.1 pM) in patients with APS and 984.5 pM (IQR: 849.0-1155.3 pM) in healthy controls (*P* = .472) (Figure 1B).

In a subanalysis comparing anticoagulant use within the APS patient cohort, FXII activation did not differ between anticoagulated patients with APS and nonanticoagulated patients with APS. Anticoagulated patients with APS used either a DOAC or VKA. Median FXIIa-AT was 30.4 pM (IQR: 24.0-46.9 pM) in anticoagulated patients with APS and 32.5 pM (IQR: 20.7-47.7 pM) in nonanticoagulated patients with APS (*P* = .429) (Figure 1C). Median FXIIa-C1Inh was 1041.3 pM (IQR: 922.3-1237.5 pM) in anticoagulated patients with APS and 1003.1 pM (IQR: 832.9-1126.0 pM) in nonanticoagulated patients with APS (*P* = .236) (Figure 1D). Factor XIIa-AT and FXIIa-C1Inh did not differ between nonanticoagulated patients with APS and healthy controls (*P* = .497 and *P* = .885, respectively).

Factor XII activation, assessed in a second subanalysis comparing APS phenotypes, did not differ between thrombotic patients with APS and obstetric patients with APS. Factor XIIa-AT was 30.4 pM (IQR: 23.6-46.5 pM) in thrombotic patients with APS and 35.1 pM (IQR: 28.6-52.4 pM) in obstetric patients with APS (*P* = .840) (Figure 1E). Factor XIIa-C1Inh was 1030.0 pM (IQR: 874.7-1231.3 pM) in thrombotic patients with APS and 1003.1 pM (IQR: 878.5-1123.0 pM) in obstetric patients with APS (*P* = .480) (Figure 1F).

In a post hoc analysis, FXII activation was increased in patients with APS and SLE. Median FXIIa-AT was 46.9 pM (IQR: 26.0-217.3 pM) in patients with APS and SLE (*n* = 11), 30.8 pM (IQR: 23.3-42.7 pM) in patients with primary APS (*n* = 58), and 30.5 pM (IQR: 24.7-66.8 pM) in healthy controls (*P* = .037 for APS with SLE vs primary APS, and *P* = .262 for APS with SLE vs healthy controls). Median FXIIa-C1Inh was 1314.2 pM (IQR: 1078.6-1654.9 pM) in patients with APS and SLE, 995.5 pM (IQR: 857.0-1126.0 pM) in patients with primary APS, and 984.5 pM (IQR: 849.0-1155.3 pM) in healthy controls (*P* < .001 for APS with SLE vs primary APS, and *P* = .018 for APS with SLE vs healthy controls).

### 3.2 FXI and FIX activation

**Figure 2:**
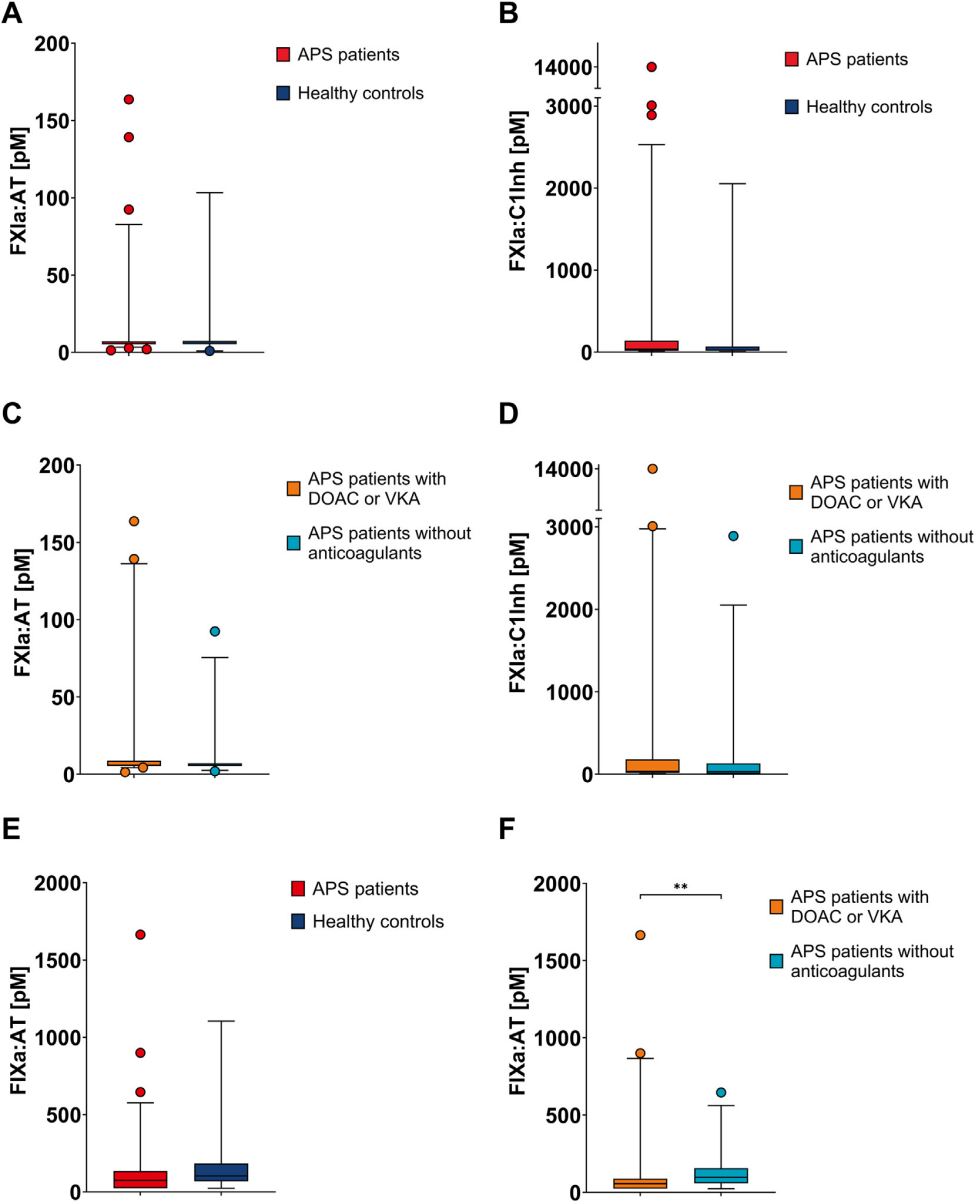
Factor (F)XI and IX activation. Activated FXI was determined in complex with antithrombin (FXIa-AT) (A) and C1-esterase inhibitor (FXIa-C1Inh) (B) in patients with antiphospholipid syndrome (APS; *n* = 73) and healthy controls (*n* = 19). A subanalysis measured FXIa-AT (C) and FXIa-C1Inh (D) in patients with APS using direct oral anticoagulants (DOACs) or vitamin K antagonists (VKAs; *n* = 40) and in patients with APS without anticoagulants (*n* = 32). Activated FIX was measured in complex with antithrombin (FIXa-AT) (E) in patients with APS (*n* = 73) and healthy controls (*n* = 19). A subanalysis determined FIXa-AT (F) in patients with APS using anticoagulants, DOAC or VKA (*n* = 40), and in patients with APS without anticoagulant medication (*n* = 32). Data are expressed as median with interquartile range. For statistical analysis, the data were log-transformed and analyzed using an independent *t*-test, ∗∗*P* ≤ .010.

Factor XI activation did not differ between patients with APS and healthy controls. Median FXIa-AT was 5.7 pM (IQR: 5.2-7.2 pM) in patients with APS and 5.9 pM (IQR: 5.2-7.4 pM) in healthy controls (*P* = .862) (Figure 2A). Median FXIa-C1Inh was 35.5 pM (IQR: 14.5-138.4 pM) in patients with APS and 24.4 pM (IQR: 16.8-69.2 pM) in healthy controls (*P* = .532) (Figure 2B).

In a subanalysis comparing anticoagulant use within the APS patient cohort, FXI activation did not differ between anticoagulated patients with APS and nonanticoagulated patients with APS. Median FXIa-AT was 5.8 pM (IQR: 5.2-8.7 pM) in anticoagulated patients with APS and 5.7 pM (IQR: 5.2-7.0 pM) in nonanticoagulated patients with APS (*P* = .307) (Figure 2C). Median FXIIa-C1Inh was 35.3 pM (IQR: 16.3-178.4 pM) in anticoagulated patients with APS and 32.9 pM (IQR: 6.5-131.3 pM) in nonanticoagulated patients with APS (*P* = .504) (Figure 2D).

Factor IX activation also did not differ between the APS study population and healthy controls when comparing the entire study cohort. Median FIXa-AT was 73.7 pM (IQR: 23.5-134.3 pM) in patients with APS and 103.0 pM (IQR: 67.8-183.8 pM) in healthy controls (*P* = .306) (Figure 2E). In a subanalysis comparing anticoagulant medication within the APS study population, median FIXa-AT was 55.6 pM (IQR: 23.5-88.1 pM) in anticoagulated patients with APS and 101.3 pM (IQR: 62.3-173.8 pM) in nonanticoagulated patients with APS (*P* = .010) (Figure 2F).

## 4 Discussion

To investigate the role of the intrinsic pathway in APS pathogenesis, activation of intrinsic coagulation factors was assessed in patients with APS and healthy controls. This study shows that, during stable disease, there is no difference in *in vivo* intrinsic pathway activity between patients with APS and healthy controls. At group level, patients with APS without recent thrombosis do not have increased FXII activation compared with healthy controls. In line with this primary finding, FXI and FIX activation did not differ between patients with APS and healthy controls.

A proportion of patients with APS in the study cohort had a history of thrombosis and received anticoagulant medication. Anticoagulant treatment dampens the intrinsic pathway through the feedback loop, by inhibiting thrombin formation. Thus, anticoagulant treatment in part of our cohort theoretically could have dampened the intrinsic pathway activation, which would otherwise have been increased. This should not affect FXII. Nevertheless, to investigate any distorting effect of anticoagulant treatment, we performed a comparison within the APS group between anticoagulated and nonanticoagulated patients, and this showed no difference in FXII activation. This suggests that anticoagulant treatment was not mitigating any unobserved but existing increased FXII activation in our patients with APS. The same was observed for FXI activation. As expected, FIX activation was decreased in patients with APS using DOAC or VKA compared with that in patients with APS without anticoagulant treatment. The mechanisms of action for these anticoagulant treatments are different but both result in decreased activated FIX. DOACs indirectly impact FIX activation through inhibition of FXa or thrombin leading to an absence of positive feedback to activate FXI, reducing FIX activation [24]. VKAs directly affect FIX activation through inhibition of FIX γ-carboxylation [25].

A prevailing concept in the pathophysiology of APS is the second hit hypothesis, in which pre-existing circulating antiphospholipid antibodies (first hit) render patients in a hypercoagulable state, and a second trigger is needed to initiate coagulation and result in a thrombotic event. Our data do not show increased intrinsic pathway activation in patients with APS at a point in time where there is no thrombosis, which could mean that the presumed NETosis in APS does not translate into increased FXII activation and downstream thrombin generation. The absence of difference in intrinsic pathway activation between patients with APS and healthy controls in our study might be explained by the lack of active thrombotic events. We speculate that, beyond the scope of this study, intrinsic pathway activation is increased upon the second hit, for instance, involving NETosis, and is thus increased only during a thrombotic event. However, this is contrary to a report observing increased NETs and elevated levels of circulating cell-free DNA in patients with APS without an active thrombotic event [26].

Interestingly, in a post hoc analysis we did find evidence of an increased *in vivo* FXII activation in the subgroup of patients with APS and SLE in our cohort. Although this was a post hoc analysis and this was not the focus of this study, these findings merit confirmation in other cohorts, comprising patients with SLE, both with and without APS. Whether an increased FXII activation in patients with SLE might reflect increased NET activation, as is observed in SLE [27], is another research topic of potential interest.

Our study included a relatively large number of patients with APS. The ELISAs performed in this study are a novel approach to measure specific complexes of *in vivo*–activated coagulation factors. Differences in APS phenotype led to anticoagulant prescription in part of the APS group. Although anticoagulated patients had no different FXII activation compared with nonanticoagulated patients, there is a theoretical possibility of confounding by indication which our study was not designed to address. Regarding the study population, a substantial proportion of patients endured only obstetric manifestations, which does not have a purely thrombotic pathophysiology. However, the majority of included patients with APS experienced thrombotic complications. Moreover, when investigating FXII activation in the subgroup with thrombotic APS, we found no difference in FXII activation compared with patients with obstetric APS.

In conclusion, patients with APS without recent thrombotic or obstetric events have similar intrinsic pathway activation to healthy controls. Further investigations into the relative contribution of the intrinsic pathway during an active thrombotic event, in anticipation of the novel FXI and FXII inhibiting agents, could, nevertheless, be of great value to this difficult to treat thrombosis population.
